# Five-point Likert scaling on MRI predicts clinically significant prostate carcinoma

**DOI:** 10.1186/s12894-015-0087-5

**Published:** 2015-09-04

**Authors:** Taisuke Harada, Takashige Abe, Fumi Kato, Ryuji Matsumoto, Hiromi Fujita, Sachiyo Murai, Naoto Miyajima, Kunihiko Tsuchiya, Satoru Maruyama, Kohsuke Kudo, Nobuo Shinohara

**Affiliations:** Department of Urology, Hokkaido University Graduate School of Medicine, North-15, West-7, North Ward, Sapporo, 060-8638 Japan; Department of Diagnostic and Interventional Radiology, Hokkaido University Hospital, Sapporo, Japan; Department of Surgical Pathology, Hokkaido University Hospital, Sapporo, Japan

## Abstract

**Background:**

To clarify the relationship between the probability of prostate cancer scaled using a 5-point Likert system and the biological characteristics of corresponding tumor foci.

**Methods:**

The present study involved 44 patients undergoing 3.0-Tesla multiparametric MRI before laparoscopic radical prostatectomy. Tracing based on pathological and MRI findings was performed. The relationship between the probability of cancer scaled using the 5-point Likert system and the biological characteristics of corresponding tumor foci was evaluated.

**Results:**

A total of 102 tumor foci were identified histologically from the 44 specimens. Of the 102 tumors, 55 were assigned a score based on MRI findings (score 1: *n* = 3; score 2: *n* = 3; score 3: *n* = 16; score 4: *n* = 11 score 5: *n* = 22), while 47 were not pointed out on MRI. The tracing study revealed that the proportion of >0.5 cm^3^ tumors increased according to the upgrade of Likert scores (score 1 or 2: 33 %; score 3: 68.8 %; score 4 or 5: 90.9 %, *χ*^2^ test, *p* < 0.0001). The proportion with a Gleason score >7 also increased from scale 2 to scale 5 (scale 2: 0 %; scale 3: 56.3 %; scale 4: 72.7 %; 5: 90.9 %, *χ*^2^ test, *p* = 0.0001). On using score 3 or higher as the threshold of cancer detection on MRI, the detection rate markedly improved if the tumor volume exceeded 0.5 cm^3^ (<0.2 cm^3^: 10.3 %; 0.2-0.5 cm^3^: 25 %; 0.5-1.0 cm^3^: 66.7 %; 1.0 < cm^3^: 92.1 %).

**Conclusions:**

Each Likert scale favobably reflected the corresponding tumor’s volume and Gleason score. Our observations show that “score 3 or higher” could be a useful threshold to predict clinically significant carcinoma when considering treatment options.

## Background

In the treatment of prostate carcinoma, one of the major disadvantages of preoperative MRI is the lack of a standardized reporting system. Sometimes, the report may be written in a narrative manner, making it difficult to compare studies from different centers. Recently, the European Society of Urogenital Radiology recommended the use of the MR Prostate Imaging Reporting and Data System (PI-RADS) [1], and the Prostate Diagnostic Imaging Consensus Meeting panel proposed using 5-point Likert scaling [2]. Regarding the 5-point Likert scaling, it was the most commonly used method in previous clinical studies [3–5]. In order to promote widespread use of the 5-point Likert scaling system in daily clinical practice, we consider that studies comparing the probability of cancer scaled using the 5-point Likert system and the biological characteristics of correspondig tumor foci should be accumulated, and the results should be shared among surgeons and radiologists as a common language.

In the present study, in order to gain further insight into the association between tumor-foci characteristics and the 5-point Likert score, we performed a detailed comparison between all small cancerous foci on whole-mount histopathology and radiological findings according to 5-point Likert scaling using the recent 3.0-Tesla multiparametric MRI.

## Methods

The study protocol was approved by the institutional review board of Hokkaido University Hospital, and patient consent was not deemed necessary because data were obtained from medical charts and patient identifying information was anonymized before analysis.

### Patients

Between June 2009 and April 2013, 80 patients with localized prostate carcinoma underwent laparoscopic radical prostatectomy (LRP). Of these, 56 patients underwent 3.0-Tesla multiparametric MRI before surgery. Excluding 12 patients due to a lack of dynamic contrast-enhanced (DCE) MRI (*n* = 5), susceptibility artifacts on diffusion- weighted imaging (DWI) (*n* = 4), or sequence differences (*n* = 3), the final study population consisted of 44 patients. No prior radiation therapy and/or androgen deprivation therapy was performed in our cohort.

### MRI technique

MRI was performed before biopsy in 6 patients, and the interval between biopsy and MRI was a median of 99 days (range: 27–855) in the remaining 38 patients.

Patients underwent MRI with a 3.0-T scanner (Achieva 3.0-T TX series R3.21; Philips Medical Systems, Best, the Netherlands) and a pelvic phased-array coil (32-channel SENSE Torso/Cardiac Coil). No endorectal coil was used. The slice thickness of all the sequences was 3 mm. The following MR sequences were obtained: axial T1-weighted image (T1WI), axial T2-weighted image (T2WI), axial, sagittal, and coronal fat-suppressed T2WI, axial DWI, and DCE MRI. DCE MRI was performed in the axial plane with one pre-contrast and four post-contrast dynamic series after the intravenous administration of gadolinium-based contrast material at a dose of 0.1 mmol per kilogram body weight and subsequent flushing with standardized 20-mL saline. The apparent diffusion coefficient (ADC) values were calculated from two DWI scans acquired with b = 0 and 2,000 s/mm2, and ADC maps were then rebuilt by calculating the ADC values in each pixel of each slice. In the present study, we did not perform any bowel preparation, or administer any agent to suppress bowel peristalsis.

### Image analysis

For this retrospective analysis, all MR images were reviewed in consensus by two radiologists with 13 and 5 years’ experience in prostate MRI. Both readers were aware that the patients had prostate cancer documented by biopsy and had undergone LRP, but were blind to all other clinical data. On pre-contrast DCE MRI, high-signal-intensity areas were considered hemorrhagic after prostate biopsy. On T2WI, a finding suggesting prostate cancer was defined as a hypointense area in the prostate compared to the adjacent parenchyma, excluding an area of hemorrhage. In the case of a wedge-shaped hypointense area without a mass effect around the adjusting prostatic tissue, we suspected chronic prostatitits rather than prostatic cancer, considering the results of other sequences together. On DWI, it was defined as a focal hyperintense area at b = 2,000 s/mm^2^ of DWI, with a low focal ADC value compared to that of the adjacent normal parenchyma on ADC mapping. On DCE-MRI, it was defined as an early enhancing area in the prostate compared to adjacent normal parenchyma. In the case of biopsy–related hemorrhage, we tried to identify an enhanced lesion by carefully comparing pre and post images. The likelihood of the presence of prostate cancer was determined based on an overall combination of the findings from T2WI, DWI, and DCE-MRI using a Likert scale between 1 and 5 (1, very low level of suspicion; 2, low level of suspicion; 3, equivocal; 4, cancer probable; 5, definitely cancer).

### Pathology

Following LRP, the specimens were fixed overnight in 10 % neutral buffered formaldehyde. After staining of the surface, each specimen was cut into transverse 5-mm-thick slices, vertical to the dorsorectal surface, and the apex and base were sagittally sectioned. After pathological evaluation, all tumor foci were mapped on the macroscopic photographs. For all LRP specimens, the primary and secondary Gleason grades as well as the combined Gleason score were recorded. The T-category was defined according to the 2009 TNM classification.

**Fig. 1:**
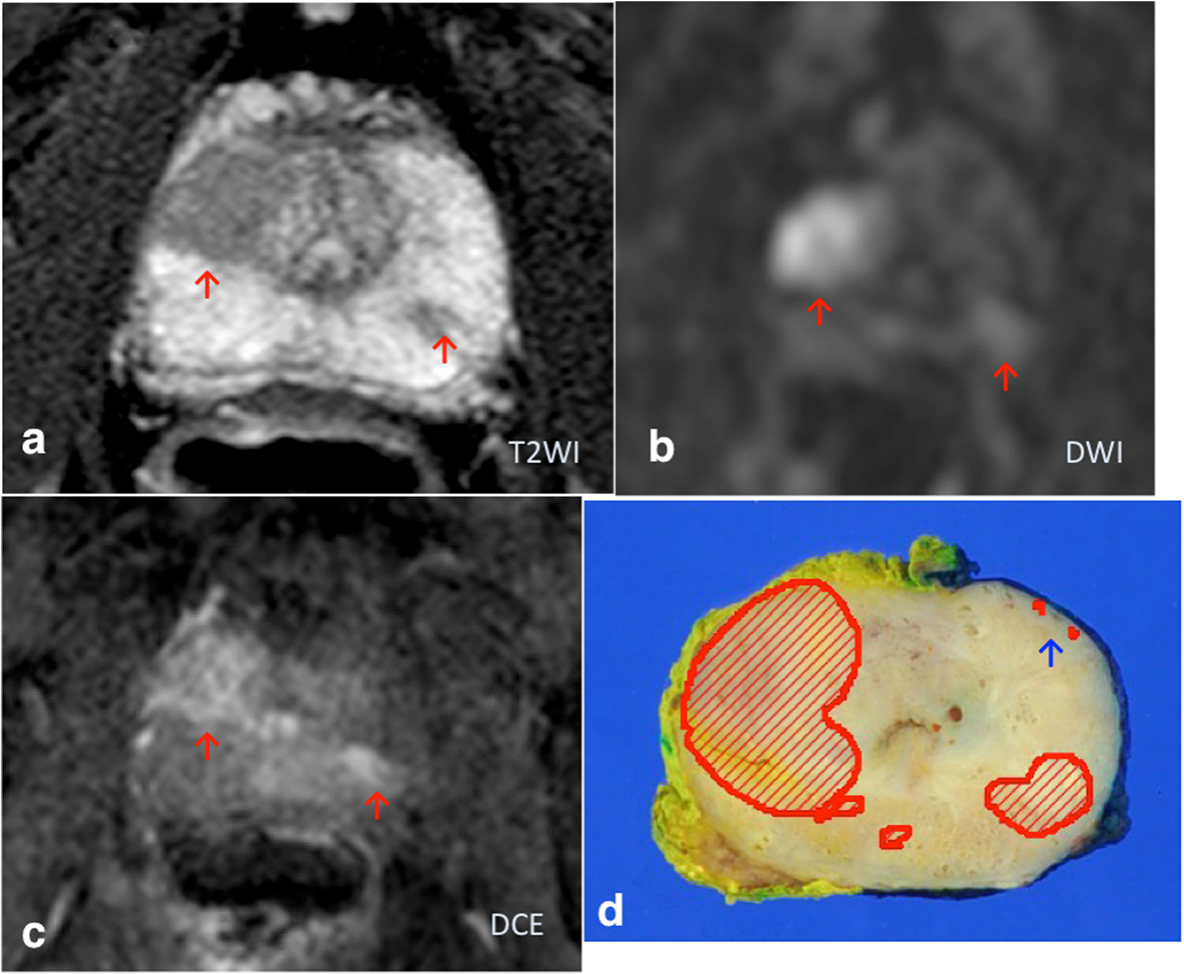
A 74-year-old patient with a PSA level of 16.08 ng/mL. A T2-weighted image (T2WI) demonstrates decreased signal intensity lesions in both lobes (**a**). These lesions show a focally increased signal intensity on diffusion-weighted (DWI, **b**) and dynamic contrast-enhanced (DCE, **c**) MRI. Using the five-point Likert scale, the two readers assigned a score of 5 to both lesions. On the other hand, the small tumor foci pointed out by the blue arrow in the histology (**d**) was not pointed out on MRI reading

For the present study, tumor areas in the macroscopic photographs were traced and measured using computer planimetry software (Image J, free software). If the distance between two tumor foci was greater than 5 mm, they were considered separate. The tumor volume was calculated by multiplying the total tumor surface area by the section thickness (5 mm). For analysis, a shrinkage factor of 1.15 was considered [6]. When the Gleason score in each focal lesion had not been described in the original pathological report, we evaluated it (HF). Thereafter, tracing based on pathological mapping and MRI findings was performed jointly by three of the authors (TH, TA, and FK; Fig. 1).

### Statistical analysis

Analyses were performed with JMP® version 11 (SAS Institute, Japan). We used the *χ*^2^ test to compare categorical characteristics among the groups. Univariate and multivariate logistic regression analyses were used to indentify characteristics associated with the visibility of cancerous lesions on 3-Tesla MRI. A p-value <0.05 was considered significant.

## Results

Clinical and pathological characteristics of the 44 patients were as follows: mean age, 67.1 years, median age, 68 years (range: 52–77); median PSA, 6.91 ng/mL (range: 1.75–37.6); median time between MRI and LRP, 10 days (range: 1–164). Thirty-one patients were in stage pT2 and 13 in pT3. Regarding the node status, all patients showed pN0. Table 1 summarizes the characteristics of tumor foci. A total of 102 tumor foci were identified histologically from the 44 LRP specimens. The median tumor volume was 0.386 cm^3^ (range: 0.0023–14.0) after shrinkage correction. The Gleason score distribution was 6 in 49, 7 in 40, and 8–9 in 13.

**Table 1:** Summary of characteristics of tumor foci

Tumor location, n	
Peripheral zone	85
Central/Transition zone only	17
Tumor volume, cm^3^	Median, 0.386 (range, 0.0023–14.0)
Tumor volume distribution, n	
<0.2 mL	39
0.2–0.5	16
0.5–1.0	9
>1.0	38
Gleason score distribution, n	
6	49
7	40
8–9	13

**Fig. 2:**
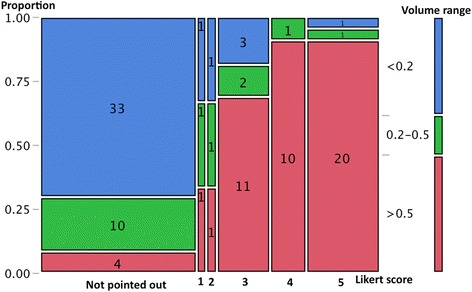
Mosaic plot of relationship between tumor volume and each assigned Likert score. The proportion of >0.5 cm^3^ tumors increased according to the upgrade of Likert scores (*χ*2 test, *p* < 0.0001)

**Fig. 3:**
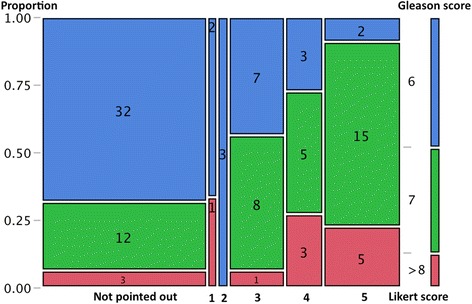
Mosaic plot of relationship between Gleason score and each assigned Likert score. The proportion of Gleason score >7 also increased from scale 2 to scale 5 (*χ*2 test, *p* = 0.0001)

Of the 102 tumors, 55 were assigned a score based on MRI findings (score 1: *n* = 3; score 2: *n* = 3; score 3: *n* = 16; score 4: *n* = 11 score 5: *n* = 22) and 47 were not pointed out on MRI. Figure 2 shows a mosaic plot of the relationship between the tumor volume and each assigned Likert score. The proportion of >0.5 cm^3^ tumors increased with the upgrade of Likert scores (score 1 or 2: 33 %; score 3: 68.8 %; score 4 or 5: 90.9 %, *χ*^2^ test, *p* < 0.0001). The median tumor volume according to each scale was 0.459 cm^3^ (range: 0.0696–0.597) for scale 1, 0.267 cm^3^ (range: 0.0558–0.954) for scale 2, 0.816 cm^3^ (range: 0.0023–5.79) for scale 3, 1.53 cm^3^ (range: 0.332–7.93) for scale 4, and 2.34 cm^3^ (range: 0.065–14.0) for scale 5. Figure 3 shows a mosaic plot of the relationship between the Gleason score and each assigned Likert　score. The proportion with a Gleason score >7 also increased from score 2 to score 5 (scale 2: 0 %; scale 3: 56.3 %; scale 4: 72.7 %; scale 5: 90.9 %, *χ*^2^ test, *p* = 0.0001).

On the other hand, 95 areas were pointed out and scaled on image review by the 2 radiologists. Tracing between pathological mapping and MRI findings revealed that 10 areas pointed out on MRI were overlapping on pathological mapping. Of the 85 non-overlapping areas, the positive predictive value for the diagnosis of cancer according to each score was 20 % (2/10) for score 1, 23.5 % (4/17) for score 2, 75 % (15/20) for score 3, 73.3 % (11/15) for score 4, and 95.7 % (22/23) for score 5. The positive predictive value was 82.8 % (48/58) for score >3.

**Fig. 4:**
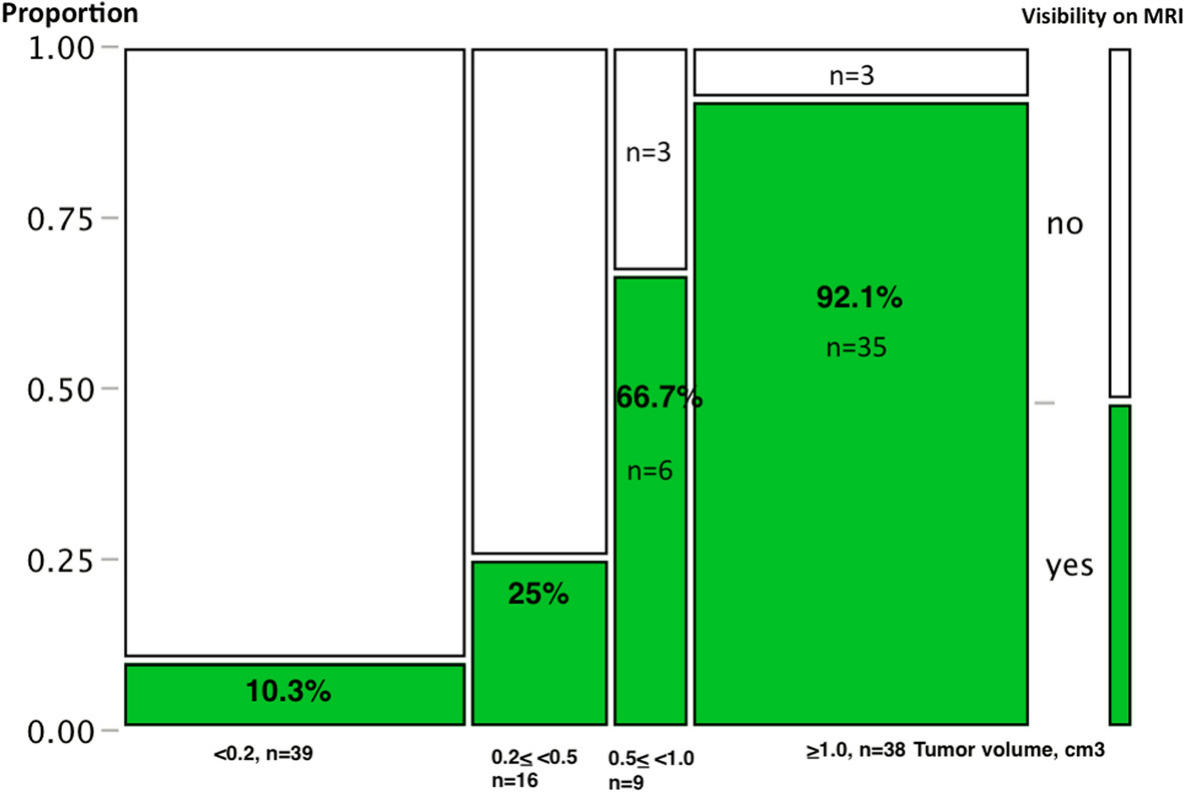
Mosaic plot of relationship between tumor visibility on 3-Tesla MRI and range of tumor volume

Based on our current observations, the assigned scores of 3–5 were treated as visible cancerous lesions on MRI in the subsequent analyses. Figure 4 shows a mosaic plot regarding tumor visibility on MRI in accordance with the tumor volume. After the tumor volume exceeded 0.5 cm^3^, the detection rate on MRI markedly improved (0.5–1.0 cm^3^: 6/9, 66.7 %, 1.0 < cm^3^: 35/38, 92.1 %). Overall, the sensitivity for cancer detection was 87.2 % (41/47) for tumors larger than 0.5 cm^3^. Table 2 shows the results of multivariate analysis to identify the characteristics associated with visibility on 3-T MRI. The tumor volume and Gleason sum were significant factors on univariate analysis. In the multivariate model, only the tumor volume remained significant.

**Table 2:** Logistic regression analysis of analysis of factors associated with visibility on 3-T MRI

		Univariate analysis		Mutivariate analysis	
Variables analyzed	No. of tumor foci	Odds ratio (95 % CI)	*p*-value	Odds ratio (95 % CI)	*p*-value
Age	102	1.03 (0.976–1.10)	0.266		
PSA	102	1.01 (0.957–1.07)	0.647		
Tumor location					
Central/Transition zone only	17	0.954 (0.329–2.73)	0.929		
Peripheral zone	85	1			
Tumor volume	102	8.53 (3.85–24.3)	<0.0001	9.24 (3.60–31.8)	<0.0001
Gleason sum	102	3.47 (1.89–7.06)	<0.0001	0.871 (0.321–2.24)	0.778

## Discussion

The usefulness of MRI for imaging prostate carcinoma has already been recognized [7]. On the other hand, most radiologists would agree that the diagnosis and localization of carcinoma are not always easy due to coexisting hyperplasia, prostatitis, or bleeding, and it is sometimes difficult to fill in medical records in a “black or white” manner. Recently, two scoring systems were recommended: PI-RADS and the Likert scale. The PI-RADS system uses multi-parametric techniques including T2-weighted imaging, DCE MRI, and DWI, and a score from one to five is given according to each variable [1]. Therefore, total scores range from 3 to 15, and a threshold of 8 or greater, or 9 or greater, has been used as a cutoff for cancer detection in previous studies [8, 9]. Regarding the Likert system, a rating from 1 to 5 was assigned based on the overall impression of MRI findings, and a threshold of 3 or higher was typically used in previous studies [3–5]. In the present study, we performed a close comparison between all small cancerous foci on whole-mount histopathology and radiological findings according to 5-point Likert scaling using the recent 3.0-Tesla multiparametric MRI. We observed that the proportion of >0.5 cm^3^ tumors increased according to the upgrade of Likert scores (score 1 or 2: 33 %; score 3: 68.8 %; score 4 or 5: 90.9 %, *χ*^2^ test, *p* < 0.0001), and the proportion of those with Gleason score >7 also increased from score 2 to score 5 (score 2: 0 %; score 3: 56.3 %; score 4: 72.7 %; 5: 90.9 %, *χ*^2^ test, *p* = 0.0001). Our observations confirmed that a threshold of 3 or higher is very helpful for clinicians when considering the possibility of significant cancer, denoting >0.5 cm^3^ or Gleason score >7 tumors. Although we did not assess PI-RADS data or inter-observer variability in scoring, Renard-Penna et al. reported favorable interobserver agreement between the Likert scale (κ = 0.80) and PI-RADS system (κ = 0.73) [8].

Regarding detectability on MRI according to the cancer volume, Ikonen S et al. previously reported that, with the use of endorectal coil 1.5-T MRI (T2-weighted), the rate of detecting carcinoma foci smaller than 5 mm was 5 %, but it was 89 % for those larger than 10 mm [10]. Roethke MC et al. reported similar results, whereby they were able to visualize 0/56 lesions with a size of <0.3 cm (0 %), 4/116 (3 %) between 0.3 and 0.5 cm, 22/169 (13 %) between 0.5 and 1 cm, 61/136 (45 %) between 1 and 2 cm, and 50/56 (89 %) >2 cm using endorectal coil 1.5-T MRI (T2-weighted) [11]. Villers et al. also reported that sensitivity, specificity, and positive and negative predictive values for cancer detection by 1.5-T pelvic phased-array coil MRI were 90, 88, 77, and 95 %, respectively, for foci larger than 0.5 cc [12]. In the present study, the positive predictive value for a diagnosis of cancer based on MRI findings was 75 % (15/20) for score 3, 73.3 % (11/15) for score 4, and 95.7 % (22/23) for score 5. Using a threshold of 3 or greater to indicate probable cancer, the detection rate on MRI markedly improved (0.5–1.0 cm^3^: 6/9, 66.7 %, 1.0 < cm^3^: 35/38, 92.1 %) when the tumor foci volume exceeded 0.5 cm^3^. Overall, sensitivity for cancer detection was 87.2 % (41/47) for tumors larger than 0.5 cm^3^, and the positive predictive value was 82.8 % (48/58) for score >3. Multivariate analysis identified only the tumor volume as being significantly correlated with visibility on MRI. Because 1 cm in diameter represents a sphere of 0.5 cm^3^, we consider that, although the detection limit of small foci was the same as that using endorectal coil or pelvic phased-array coil 1.5-T MRI, modern 3.0-Tesla multiparametric MRI offers a more sophisticated image of the prostate and can clearly visualize most tumors larger than 0.5 cm^3^. In other words, 3.0-Tesla can identify clinically significant disease in terms of a tumor volume >0.5 cm^3^. In contrast, it can barely detect tumors with a volume of less than 0.5 cm^3^ regardless of the Gleason score. In the current study, an endorectal coil was not used, and we, therefore, did not generate data on how an endorectal coil can aid in tumor depiction. Previously, Park BK et al. and Sosna J et al. reported that 3.0-T pelvic phased-array MRI could produce an image equivalent to 1.5-T endorectal MRI [13, 14]. Kim BS et al. also reported that the staging ability was not significantly different between 3.0-T pelvic phased-array MRI and 3.0-T endorectal coil MRI [15]. In contrast, Turkbey et al. recently reported that the combined use of a nonendorectal coil and an endorectal coil led to the detection of more cancerous foci than the sole use of a nonendorectal coil [16]. At present, we consider that patients’ discomfort and the additional cost are drawbacks to the use of an endorectal coil, and preoperative evaluation with a nonendorectal coil would be more acceptable universally in daily clinical practice.

As described above, the positive predictive value for a diagnosis of cancer based on MRI findings was 75 % (15/20) for score 3, 73.3 % (11/15) for score 4, and 95.7 % (22/23) for score 5. Conversely, 10 lesions assigned a score >3 (3: *n* = 5; 4: *n* = 4; 5: *n* = 1) were false-positive findings on MRI. After the analysis, we convened a meeting with the pathologists to discuss the cause of these false-positive findings, and found that the most frequent histological finding was inflammation (*n* = 7), and abscess formation was also noted in one case (data not shown). It would be optimal to perform MRI before prostate biopsy to minimize these artifacts. It has been established that an interval of at least eight weeks is needed to minimize artifacts due to prostate biopsy [17]. Regarding the 6 foci greater than 0.5 cm^3^ which were missed on MRI (Fig. 3), a strong background due to hemorrhage (*n* = 1), an anterior TZ location (*n* = 1), an apex location (*n* = 1), and islet distribution of small foci (*n* = 3) may have compromised visualization on MRI. Regarding the relationship between the tumor location and MRI visibility, previous studies showed the lack of additional benefit of DCE-MRI and the difficulty of cancer detection in the transition zone [18, 19]. In the present study, as shown in Table 2, although the tumor location was not associated with visibility on 3-T MRI, our sample size was too small to draw a definitive conclusion, and we agree that the co-existing prostatic hyperplasia might compromise cancer detection in the transition zone.

Our study has several potential limitations. Firstly, this was a small retrospective study. There was a selection bias in that only patients undergoing prostatectomy were enrolled. As described above, because all MR images were reviewed in consensus by two radiologists during a single session, we do not have data on inter- or intraobserver variability of the Likert scale. The lack of an endorectal coil may have influenced our observations. Furthermore, in cases undergoing MRI evaluation after biopsy, post-biopsy hemorrhage might have resulted in difficulty of tumor detection. It remains unknown whether the results would be reproducible in a different institute with a reader with a different experience level. Further validation studies are warranted to evaluate whether a scoring system including a Likert scale can become a common language among physicians treating prostate carcinoma patients in daily clinical practice.

## Conclusion

Each Likert scale well reflected the corresponding tumor’s volume and Gleason score. Our observations show that “scale 3 or higher” could be a useful threshold to predict clinically significant carcinoma when considering treatment options.

## Abbreviations

LRP: Laparoscopic prostatectomy
DCE-MRI: Dynamic contrast-enhanced MRI
DWI: Diffusion-weighted imaging
ADC: Apparent diffusion coefficient
